# A novel rodent *Chapparvovirus* in feces of wild rats

**DOI:** 10.1186/s12985-016-0589-0

**Published:** 2016-07-29

**Authors:** Shixing Yang, Zhijian Liu, Yan Wang, Wang Li, Xingli Fu, Yuan Lin, Quan Shen, Xiaochun Wang, Hua Wang, Wen Zhang

**Affiliations:** 1 School of Medicine, Jiangsu University, Zhenjiang, Jiangsu 212013 People’s Republic of China; 2 School of Basic Medical Sciences, Ningxia Medical University, Yinchuan, Gansu 750000 People’s Republic of China; 3 School of Medicine, Jiangsu University, 301 Xuefu Road, Zhenjiang, Jiangsu 200240 People’s Republic of China

**Keywords:** Wild rat, *Chapparvovirus*, Genome structure, Viral Metagenomics

## Abstract

*Chapparvovirus*, a recently determined new genus in the family *Parvoviridae*, can infect many species of animals including bats, chickens, and pigs. Here, using viral metagenomics method, we identified a novel *Chapparvovirus* from feces of wild rats and designated it as rat parvovirus 2 (RPV2). The nearly complete genome of RPV2 is 4222-nt long and includes two ORFs encoding a 654-aa nonstructural protein 1 (NS1) and a 472-aa capsid protein (VP), respectively. Phylogenetic analysis over the amino acid sequence of the NS1 showed that RPV2 clustered with Eidolon helvum parvovirus 2 (EHPV2), porcine parvovirus 7 (PPV7), and turkey parvovirus 1 (TP1), forming a separate clade. Sequence analysis indicated that the NS1 protein of RPV2 shared the highest amino acid sequence identity (51 %) with that of EHPV2. According to the genetic distance-based criteria, RPV2 identified here belongs to a novel species of *Chapparvovirus*.

## Findings

Parvoviruses can infect many species of animals and cause illness through individual or together with other viruses [1–3]. The virion of parvovirus is icosahedral and non-enveloped package, whose core is a linear single-stranded DNA genome of approximately 5 Kb long. The family *Parvoviridae* is divided into the subfamilies *Parvovirinae* and *Densovirinae* [4], which infect vertebrates and invertebrates, respectively. The subfamily *Parvovirinae* is divided into 8 genera: *Dependoparvovirus*, *Copiparvovirus*, *Bocaparvovirus*, *Amdoparvovirus*, *Aveparvovirus*, *Protoparvovirus*, *Tetraparvovirus*, and *Erythroparvovirus*. Recently two other genera of *Marinoparvovirus* and *Chapparvovirus* were identified. The genus of *Marinoparvovirus* only has one member named sesavirus which was discovered from the feces of a California sea lion pup suffering from malnutrition and pneumonia [5]. The genus of *Chapparvovirus* have been detected from chicken feces samples, Eidolon helvum feces samples, and porcine multiple tissue samples including serum, rectal swab, nasal swab, and lung lavage [6–8]. *Chapparvovirus,* as a novel genus of *parvovirinae,* still have unclear species. Here, using viral metagenomics and PCR amplification, we identified a novel species of *Chapparvovirus* from the feces of wild rats and characterized its nearly complete genome.

In the present study, we investigated 40 fecal samples from wild rats captured by the Chinese Center for Disease Control and Prevention in Taizhou City from three districts of Taizhou City including Taixing (*n* = 15), Gaogang (*n* = 15), and Hailing (*n* = 10) from June to August in 2004. All of the wild rats were adults and the exact age was unknown.

Viral metagenomics method was used to characterize viral sequences in the fecal samples. Fecal samples were suspended in DPBS, vortexed for 10 min, and then centrifuged at 12,000 × g for 10 min. The stool suspensions were collected in 1.5 ml centrifuge tubes. Three pools were randomly generated, each of which contained 10 fecal suspensions. After low speed centrifugation and filtration, the samples were treated with DNase and RNase, to reduce levels of mink nucleic acids while viral genomes are protected from digestion within viral capsid [9, 10]. Three libraries were then constructed using Nextera XT DNA Sample Preparation Kit (Illumina) and sequenced using the Miseq Illumina platform with 250 bases paired ends with a distinct molecular tag for each pool. Resulting raw reads were trimmed for quality and primer, and *de novo* assembled into contigs. Sequences and contigs were compared to the GenBank non-redundant protein database using BLASTx with an E-value cutoff of <10^−5^.

**Fig. 1:**
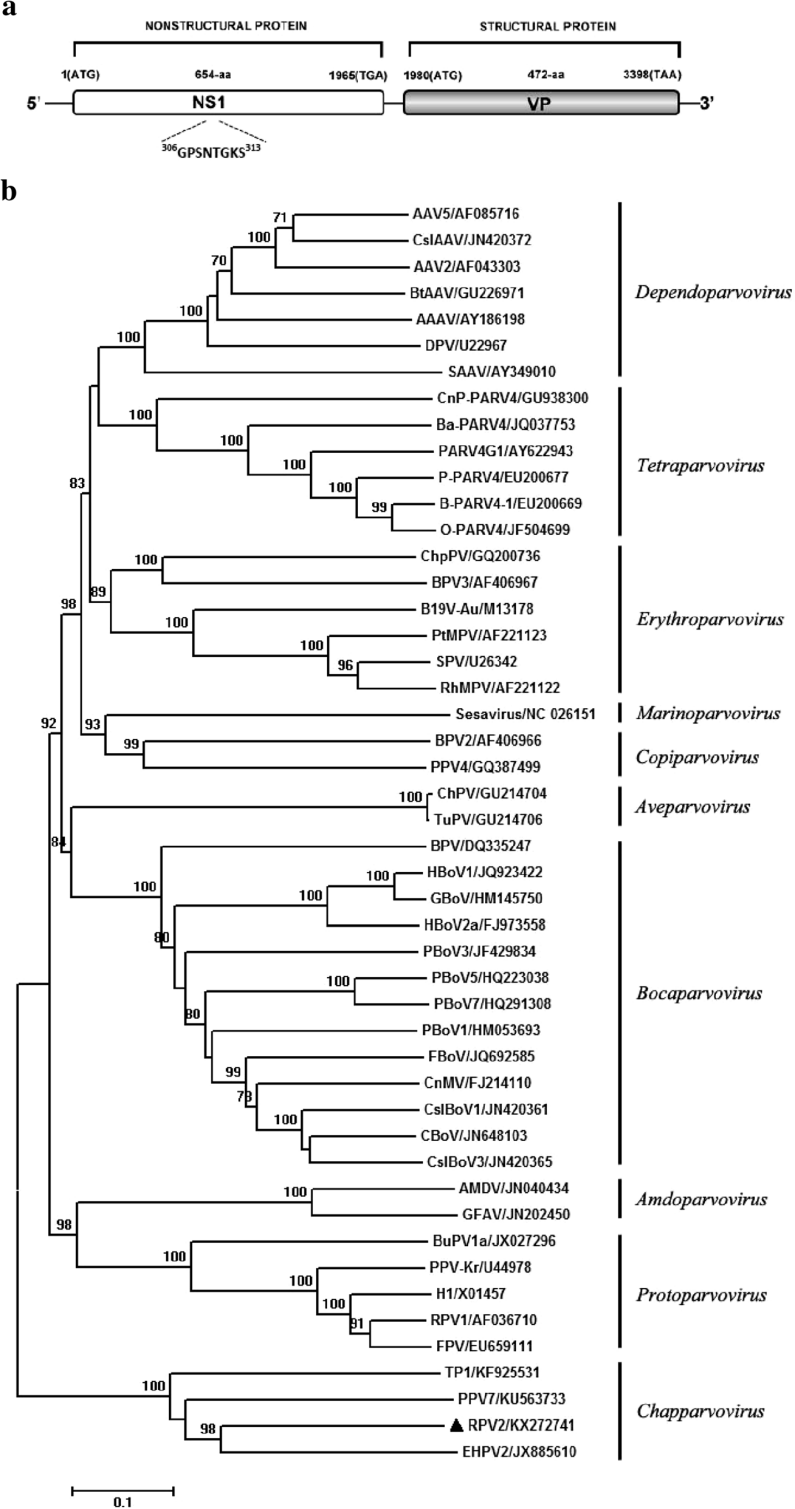
Genome organization and phylogenetic analysis of RPV2. **a** The genome rrganization of RPV2. The NS1 and VP proteins and the conservative ATP or GTP binding Walker loop motif are shown. **b** Phylogenetic tree was constructed over the NS1 amino acid sequences of 48 members in the *Parvovirinae*. The tree was derived using Neighbor-Joining analysis with 1000 bootstrap replicates. Scale bars indicate nucleotide substitutions per site, vertical bars represent the genera, and GenBank accession numbers are shown for the reference virus sequences, RPV2 identified in this study are marked with black triangle

Results indicated there were 66 sequences reads showing the best BLASTx matches to EHPV2. The nearly complete genome was then determined by filling gaps by PCR and sequenced by Sanger method. The novel *Chapparvovirus* was designated rat parvovirus 2 (RPV2) and submitted to GenBank with accession no. KX272741. The near complete genome of RPV2 consists of 4222-nt and encodes two major proteins including the NS1 protein and VP protein, respectively. The NS1 protein was 654 amino acid in length possessing the ATP or GTP binding Walker loop motif ^306^GPSNTGKS^313^ (Fig. 1, panel a). Sequence analysis indicated that RPV2 NS1 shares the highest amino acid sequence identity of 51 % with EHPV2. The VP protein was 472-aa in length and significantly shorter than other members of the subfamily *Parvoviridae,* which has an approximately 700-aa capsid protein. In addition, the conserve phospholipase A_2_ (PLA2) motif widely presented in the other members of the subfamily *Parvoviridae* is lack in RPV2 VP protein [11]. The RPV2 VP protein shares the highest amino acid sequence identity of 44 % with PPV7. The ICTV states that parvoviruses sharing >85 % amino acid identity of NS1 protein belong to the same species. Based on the criteria, RPV2 identified here belongs to a novel species of *Chapparvovirus*.

To determine the relationship between RPV2 and the other members of the subfamily *Parvoviridae*, phylogenetic analysis over the NS1 protein of 48 members in the *Parvovirinae* was performed. Amino acid sequences of NS1 were aligned by ClUSTAL W, and phylogenetic trees were constructed with MEGA 6.0 software using the Neighbor-Joining statistical method based on the Jones-Taylor-Thornton matrix-based model. Result showed that RPV2 clustered with EHPV2, PPV7, and TP1, forming a separate clade (Fig. 1, panel b). Among them, RPV2 has the closest relationship with EHPV2, which was identified from fecal samples of Eidolon helvum [7].

To investigate the prevalence of RPV2 in wild rats, viral nucleic acid was extracted from all the 40 individual samples using TaKaRa MiniBEST Universal Genomic DNA Extraction Kit Ver.5.0 (TaKaRa, Japan). A set of nested primers designed basing on the NS1 nucleotide sequence of RPV2 was used to perform PCR screening in the 40 rat fecal samples. Primer RPV2 F1 (5′-TGGGTTCAGGCTACACAGGGTGG-3′) and RPV2 R1 (5′-CAGTCGCCCCTCTCAGGGCT-3′) were used for the first round of PCR, and RPV2 F2 (5′-AGCTGTACCCTGGGGAGAAATTGT-3′) and RPV2 R2 (5′-CGGGTTGTTGTCCTCTCGGCA-3′) for the second round, the expected length of amplified fragment was 419 bp. Our results indicated 17.5 % (7/40) of the fecal samples were positive. The PCR amplicons were T-A cloned and sequenced by Sanger method. Sequence analysis showed that the seven amplicons shared >98 % nucleotide identity, indicating a single virus strain was prevalent in the wild rats in this area.

## Conclusions

In summary, we detected a novel rodent *Chapparvovirus* in wild rats and characterized its nearly complete genome. Phylogenetic analysis indicated that the RPV2 clustered with EHPV2, PPV7, and TP1, forming a separate clade. According to the genetic distance-based criteria, RPV2 belonged to a novel species of *Chapparvovirus*. Our epidemiologic data indicated RPV2 was prevalent in the wild rats in this area.

## Abbreviations

BLAST, basic local alignment search tool; DPBS, Dulbecco’s phosphate buffered saline; EHPV2, eidolon helvum parvovirus 2; NS1, nonstructural protein 1; ORF, open reading frame; PCR, polymerase chain reaction; PLA2, phospholipase A_2_; PPV7, porcine parvovirus 7; RPV2, rat *Chapparvovirus* 2; VP, capsid protein
